# Association between constant and intermittent knee pain and T2 values and cartilage thickness: data from the osteoarthritis initiative

**DOI:** 10.1186/s13075-025-03667-9

**Published:** 2025-10-22

**Authors:** Maximilian T. Löffler, Gabby B. Joseph, John A. Lynch, Nancy E. Lane, Valentina Pedoia, Sharmila Majumdar, Michael Nevitt, Charles McCulloch, Thomas M. Link

**Affiliations:** 1 Department of Diagnostic and Interventional Radiology, University Medical Center Freiburg, Freiburg im Breisgau, Germany; 2 Department of Radiology and Biomedical Imaging, UCSF, 185 Berry Street, Suite 350, Lobby 6, San Francisco, CA 94143 USA; 3 Department of Diagnostic and Interventional Neuroradiology, School of Medicine, Klinikum rechts der Isar, Technical University of Munich, Munich, Germany; 4 Department of Medicine, Center for Musculoskeletal Health, UC Davis, Health Sacramento, CA USA; 5 Department of Epidemiology and Biostatistics, UCSF, San Francisco, CA USA

**Keywords:** Knee pain, Knee osteoarthritis, Compositional MRI, Cartilage thickness, Cartilage T2 values

## Abstract

**Background:**

We investigated whether cartilage composition and thickness and its change over time were associated with future intermittent and constant knee pain.

**Methods:**

Osteoarthritis Initiative participants with 3T MRI scans from baseline to 36-month visits were selected. Outcomes were Intermittent and Constant Osteoarthritis Pain (ICOAP) scores in the right knee at the 48-month visit (0 to 100 = highest pain). We measured T_2_ values and cartilage thickness in 5 regions in the right knee from baseline to 36-months using deep-learning-based segmentation. Associations between baseline and change in cartilage biomarkers with pain scores were tested using adjusted logistic and linear regression models.

**Results:**

Of 3780 included participants, 1042(28%) had symptomatic knee OA in any knee at baseline. At 48 months, 1671(44%) had intermittent and 265(7%) constant pain in the right knee. Odds for having intermittent knee pain increased with longer baseline T_2_ values in medial and lateral femoral cartilage (OR[95%CI]: 1.05[1.02–1.08] and 1.06[1.03–1.09] for 1 ms longer) and thinner baseline patellar cartilage (0.65[0.53–0.81] for 1 mm thicker). Greater annual rates of patellar cartilage thinning were associated with higher odds of constant knee pain (93.4[7.66–1139] for 1 mm/yr greater). Among those with knee pain, greater annual rates of increase in medial and lateral tibial cartilage T_2_ led to more intermittent knee pain (percent change[95%CI]: 8.02[2.87–13.4] and 7.85[3.39–12.5] for 1 ms/yr greater). Thicker lateral tibial cartilage at baseline led to less constant knee pain (beta coeff.[95%CI]: -11.8[-19.8–3.76] for 1 mm thicker).

**Conclusions:**

Impaired femoral cartilage composition, indicated by longer T_2_ values, preceded intermittent knee pain found in early-stage OA. Constant knee pain characteristic for late-stage OA was related to greater cartilage thickness loss.

**Supplementary Information:**

The online version contains supplementary material available at 10.1186/s13075-025-03667-9.

## Introduction

Symptomatic knee osteoarthritis (OA) is defined as the presence of radiographic signs of joint degeneration and frequent knee symptoms [1] and affects an estimated 14 million people in the US [2]. Symptomatic knee OA with pain as its hallmark symptom is the leading cause of disability [3] and reduced quality of life due to mobility impairment [4]. Pain, in general, is the main reason why people see a primary care physician and knee pain can account for up to 5% of these consultations [5].

Joint pain due to OA can be measured by self-report with the visual analog scale (VAS) or more detailed rating scales. The Western Ontario and McMaster Universities Osteoarthritis Index (WOMAC) is a widely used instrument that asks about OA related pain, stiffness, and physical disability in the hip or knee. Its original purpose was to evaluate clinically important changes of symptoms as a result of treatment intervention [6]. Large scale cohort studies and clinical trials typically assess pain in the range of 3–12 months using the WOMAC or the Knee Outcomes in Osteoarthritis Survey (KOOS) [7, 8]. Between 23 and 32% of patients with knee pain reported significant variability over 72 months [9]. Variability of pain can be an elusive concept if assessed in intervals of several months using WOMAC or KOOS. Fluctuations of pain can appear in a much shorter time frame with varying intensity, frequency, and duration. Specific questions about temporal patterns of pain experience can aid in this regard. It is understood that persons with early-stage symptomatic knee OA typically present with intermittent, activity-induced knee pain, but no gold-standard definition exists [10]. The Outcome Measures in Rheumatology (OMERACT) initiative and the Osteoarthritis Research Society International (OARSI) seeking to develop a new pain metric for use in clinical practice to assess OA progression found two distinct pain types [11]. First, a quite constant and aching background pain and, second, a less frequent, more intense, and often unpredictable pain. This understanding led to the development of the Intermittent and Constant Osteoarthritis Pain (ICOAP) score for the hip or knee [12].

Radiographic knee OA is an imprecise marker of knee pain or disability [13]. The concordance of knee pain and radiographic knee OA is low in that a variable number of individuals with knee pain have radiographic signs of OA and vice versa [13]. Magnetic resonance imaging (MRI) can assess the whole joint including cartilage [14]. However, only bone marrow lesions and effusion/synovitis are clearly associated with knee pain in OA, whereas the association between cartilage defects and pain seems to be weaker [15]. Cartilage morphometry, which can be automated using cartilage segmentation algorithms [16], quantifies morphologic features and changes of cartilage thickness and volume. Several studies found associations of structural cartilage damage with knee pain [17–23].

Beyond morphology, compositional MRI, including T_2_ values, can detect cartilage damage at a pre-structural stage before cartilage lesions become manifest [24–26]. Cartilage T_2_ values are associated with knee pain [27] and have the potential to diagnose early stages of knee OA [28]. Evidence also indicated that changes in T_2_ values precede changes in cartilage thickness and defects in knee OA [29, 30]. However, knowledge from longitudinal studies about the effect of cartilage imaging biomarkers on knee pain is scarce. The purpose of this study was to investigate how changes in cartilage biomarkers precede different types of knee pain that are used to clinically distinguish earlier from later stages of knee OA. The objectives were to identify how (1) baseline values and (2) annual change in T_2_ values and cartilage thickness measured from baseline to 36 months are associated with intermittent and constant knee pain assessed at 48 months.

## Methods

### Study population and design

**Fig. 1:**
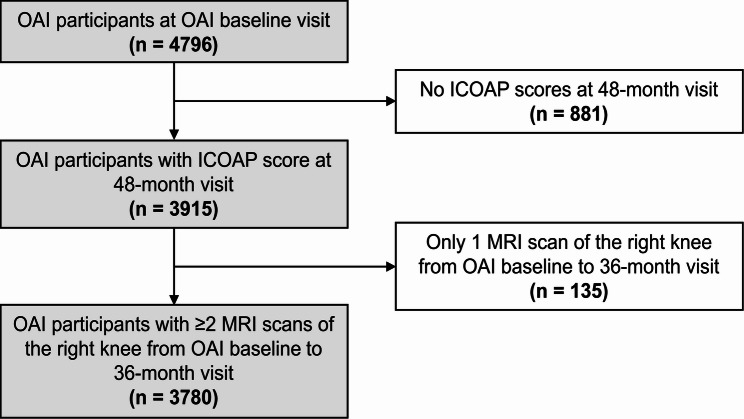
Subject selection from the OAI cohort

For this study, we selected all participants from the Osteoarthritis Initiative (OAI) who had ICOAP scores of the right knee available at the 48-month visit (*n* = 3915). Furthermore, we included only participants with ≥ 2 MRI scans at the baseline, 12-month, 24-month, or the 36-month visit that were required to calculate linear change in MRI-based cartilage biomarkers (*n* = 135, 3.4%, excluded). A total of 3780 participants were analyzed in this study. A subject selection flowchart is shown in Fig. 1.

The OAI is a multicenter, longitudinal, observational cohort study investigating biomarkers that relate to the onset and progression of OA in 4796 individuals (https://nda.nih.gov/oai/). Participants were assigned into an *incidence* (*n* = 3,284), *progression* (*n* = 1,390), or *control subcohort* (*n* = 122) at enrollment reflecting the full spectrum of the disease [1, 7]. Participants in the *progression subcohort* had symptomatic femorotibial OA in at least one knee at baseline. Symptomatic OA is defined by the presence of frequent symptoms (pain, aching, or stiffness more than half the days of a month for the past 12 months) in a joint with femorotibial radiographic OA. This definition of frequent knee symptoms is derived from the 1981 American College of Rheumatology (ACR) clinical criteria for the diagnosis of knee OA [31]. Radiographic knee OA is defined as definite osteophytes with or without joint space narrowing equal to a Kellgren-Lawrence (KL) grade 2 or higher [32]. The *incidence subcohort* comprises individuals with known and putative risk factors for developing symptomatic knee OA, but without symptomatic knee OA in either knee at baseline. The *control subcohort* includes individuals with neither frequent knee symptoms, radiographic signs of, nor risk factors for femorotibial OA in either knee.

### Ethics approval

The National Institute of Arthritis and Musculoskeletal and Skin Diseases (NIAMS) at the National Institutes of Health (NIH) appointed an independent Observational Study Monitoring Board (OSMB) to oversee the OAI study from 2002 to 2014. The OSMB’s responsibilities included protecting the safety of study participants, considering ethical and other external factors that could impact participant safety, and monitoring the progress of the study. This Health Insurance Portability and Accountability Act (HIPAA)-compliant study was approved by the OSMB and written informed consent was obtained from all study participants at each institution.

### Clinical data and radiographs

We collected baseline data, including age, sex, body mass index (BMI), the Physical Activity Scale of the Elderly (PASE) score, race, and subcohort assignments. The PASE is an established and reliable questionnaire for the assessment of physical activity used in prior work [33]. Standardized bilateral standing posterior-anterior fixed flexion knee radiographs were acquired in all participants in the OAI. To characterize baseline disease burden, knee Kellgren-Lawrence gradings from the OAI baseline visit were scored as previously described [34].

### Pain data

We used ICOAP scores in the right knee as the outcome that was assessed at the 48-month visit. The ICOAP score for hip and knee OA was developed in an OMERACT/OARSI initiative for use by clinicians to monitor the development of pain during OA progression and in response to therapy and to find indications that could trigger referral to a surgeon for consideration of joint replacement [12]. This 11-item self-administered questionnaire assesses constant pain and pain that comes and goes (= intermittent pain) that was experienced in the past week. For both pain types, individual items assess pain intensity, effect on sleep, on quality of life, extent to which the pain “frustrates or annoys”, and “worries or upsets”. Frequency of intermittent pain is assessed in an additional item. Each item allows answers on the ordinal scale from 0 = not at all/never to 4 = extremely/very often. Final scores for constant or intermittent pain constitute sums of individual items that are scaled to a range of 0–100, with 0 being no pain and 100 being worst pain. The ICOAP measure showed excellent test-retest reliability [12]. We defined the presence of intermittent pain and constant pain as intermittent and constant pain scores >0, respectively.

### MR imaging

Imaging biomarkers (T_2_ values and cartilage thickness) derived from MRI scans at baseline, 12-month, 24-month, and 36-month visits were used as predictors. MR images were acquired on four identical 3 T scanners (Magnetom Trio; Siemens Healthineers, Erlangen, Germany) using quadrature transmit-receive coils (USA Instruments, Aurora, OH, USA). From the OAI imaging protocol, we used sagittal 3D dual-echo in steady state (DESS; TE/TR = 4.7/16.3 ms, flip angle = 25°) and sagittal T_2_ multi-slice multi‐echo (MSME) spin‐echo sequences (TE/TR = 10, 20, 30, 40, 50, 60, 70/2700 ms) acquired in the right knee [35].

#### T_2_ values

As a biomarker of cartilage composition [26], T_2_ values were automatically measured in knee subregions in MSME sequences. The segmentation and T_2_ quantification pipeline is described in Razmjoo et al., with a figure provided in that publication (see Fig. 1) [36]. In brief, cartilage was fully automatically segmented in five cartilage regions using a 3D V-Net deep-learning architecture and the Sørensen–Dice coefficient as the main loss function to solve the semantic classification problem as suggested by Milletari et al. [37]. This resulted in mean cartilage T_2_ values in five regions of all analyzed knees: Medial femoral (MF), lateral femoral (LF), medial tibial (MT), lateral tibial (LT), and patellar (PAT). Trochlea cartilage segmentations were not available. The accuracy of extracted T_2_ values and of segmentation masks (measured as dice coefficients) was uncorrelated in most cases, with the least correlations in the medial femoral region [36].

#### Cartilage thickness

**Fig. 2:**
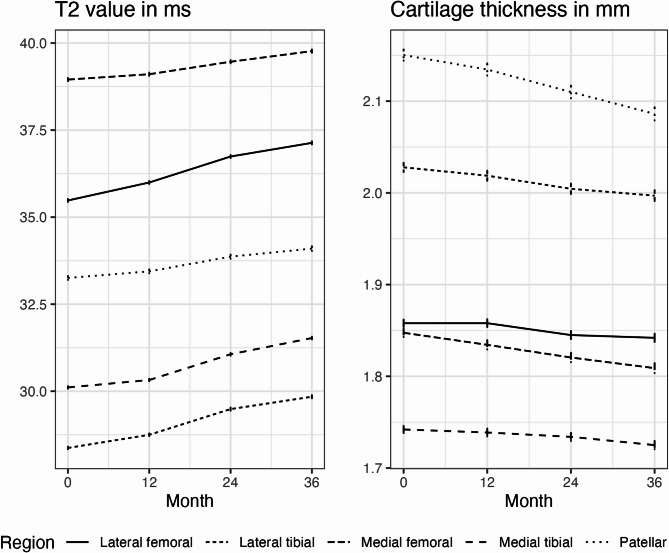
Mean and standard error of T_2_ values and cartilage thickness between baseline and 36 months

A fully-automatic deep-learning-based method for knee cartilage segmentation and thickness measurements in subregions was previously developed and validated as described by Iriondo et al., with figures provided in that publication (see Figures 2 and 3) [38]. An ensemble of six convolutional neural networks [37] (three 3D VNet and three 2D UNet-like architectures) were trained to segment femoral, tibial, and patellar cartilage in DESS sequences. A subsegmentation algorithm divided these regions into medial and lateral regions of the femur and tibia, respectively. In the femur, central weight-bearing regions of the medial and lateral femoral condyles were isolated for cartilage thickness measurements according to a rule described in Glaser et al. [39]. Euclidian distance transform and skeletonization were performed per sagittal slice in each region. Mean cartilage thickness was calculated as the average of distances along each point on the skeleton line. This resulted in mean cartilage thickness measurements in the same five regions as for T_2_ values, except for central weight-bearing regions only in the femur. The accuracy of automatic cartilage thickness measurements was good compared to manual measurements, with the upper 95% CI for the largest mean difference within 0.15 mm, which is less than half the in-plane pixel resolution [38].

### Statistical analysis

Statistical analyses were performed using R-Studio (version 2023.12.1, Posit Software PBC, Boston, MA) and SPSS Statistics (version 29, IBM, Armonk, NY). Descriptive statistics of baseline characteristics (age, sex, race, BMI, PASE score, subcohort membership, and Kellgren-Lawrence grade) were calculated for participants with any intermittent, any constant, or without pain at 48 months.

We used the “growth curve method” to calculate associations between ICOAP scores at 48 months (= fixed outcome) and MRI-based cartilage biomarkers measured at baseline, 12-, 24-, and 36-month visits (= longitudinally measured predictor) [40]. Therefore, slope and intercept of T_2_ values and cartilage thickness were calculated in individual linear regression models fit to the longitudinal data of each subject. We chose a linear model because of only 4 samples and to reduce issues of multicollinearity. Plots of longitudinal data confirmed linear change (Fig. 2). The intercept represents the predicted value at baseline and the slope represents the annual rate of change during the 3-year period. Baseline values and annual rates of change of cartilage biomarkers were entered in all statistical models as predictors.

Because of a zero-inflated distribution of ICOAP scores a two-part model [41] was employed to avoid violation of the normality assumption and to preserve information of non-dichotomous scores. Further, we assumed different effects of the longitudinally measured predictors (baseline value and annual change) on dichotomous and non-dichotomous outcomes. First, intermittent and constant pain scores, respectively, were dichotomized to the presence of no pain (score = 0) and any pain (score >0). Odds ratios (OR) and 95% confidence intervals (CIs) for the presence of intermittent or constant pain (= dichotomized outcomes) for one unit change in baseline value or annual change of cartilage biomarker (= predictors) were calculated in logistic regression models. Second, beta coefficients and 95% CIs for ICOAP scores (= continuous outcomes) were calculated in linear regression models only for participants with any intermittent or constant pain (score >0), respectively. Beta coefficients represent the change in constant pain score for one unit change in baseline value or annual change of cartilage biomarker. Normal distribution of residuals was analyzed in P–P plots. Natural logarithmic transformation of intermittent pain scores was required to yield normally distributed residuals. Thus, change in intermittent pain score is approximated by the formula $\:percent\:change\:\approx\:100\times\:({e}^{Beta\:coeff.}-1)$. This represents the percent change in intermittent pain score for one unit change in baseline value or annual change of cartilage biomarker. In a secondary analysis, we investigated the association of T_2_ values with cartilage thickness and, in particular, if higher T_2_ values at baseline predicted cartilage loss over time.

All models were adjusted for age, sex, subcohort membership, baseline BMI, and baseline PASE score, as these factors are known to influence OA and are expected to affect pain perception [42]. As previously described, the three subcohorts were defined either by the absence of symptomatic OA in any knee and the presence of known risk factors for knee OA (incidence), or the presence of symptomatic OA in one or both knees (progression), or none of the above (control). Thus, adjusting for subcohort membership considers the presence or absence of frequent knee symptoms (pain, aching, or stiffness) at baseline as included in the definition of symptomatic knee OA. We chose this approach to reduce confounding of baseline pain because ICOAP scores were not available at baseline. In a sensitivity analysis, we adjusted our models for baseline WOMAC pain instead of subcohort membership in order to compare both approaches.

False discovery rate (FDR, q-value) as proposed by Benjamini and Hochberg was used to control Type I error in multiple comparisons (2 outcomes [intermittent and constant pain] and 20 predictors [slope and intercept of T_2_-values and cartilage thickness in five regions each] = 40 tests). A q-value threshold of 0.05 defined statistical significance, except for the secondary analysis where a standard p-level of 0.05 defined statistical significance.

## Results

### Subject characteristics and outcomes

The study sample consisted of 3780 participants (57% female subjects, *n* = 2170) that had ICOAP scores available at the 48-month visit and at least two MRI scans at the baseline, 12-month, 24-month, or 36-month visit. Baseline data of the study sample stratified by the presence of any intermittent or any constant knee pain are shown in Table 1. Three (0.1%) persons had missing BMI and 21 (0.6%) persons had missing PASE scores at baseline. 1671 (44%) persons had any intermittent pain and 276 (7%) had any constant pain in the right knee (scores > 0) at 48 months. Persons with any intermittent or constant pain had a mean score of 19.2 ± 17.0 or 22.3 ± 25.3, respectively.

**Table 1:** Baseline characteristics of 3780 subjects stratified by the presence of any intermittent or any constant pain at 48 months

	All	Any intermittent pain	Any constant pain
N	3780	2109	447
Age, years, mean (SD)	61.0 (9.1)	60.3 (8.9)	58.7 (8.4)
Sex, n of female subjects (%)	2170 (57%)	1227 (58%)	263 (59%)
Body mass index, kg/m^2^, mean (SD)	28.5 (4.8)	29.0 (4.7)	30.5 (5.1)
PASE, mean (SD)	164.3 (81.9)	167.1 (83.3)	173.2 (87.1)
Race, n (%)			
White	3076 (81%)	1712 (81%)	280 (63%)
Black/African American	618 (16%)	346 (16%)	157 (35%)
Asian	29 (1%)	15 (1%)	1 (0%)
Other non-White	53 (1%)	34 (2%)	9 (2%)
Subcohort, n (%)			
Incidence	2625 (69%)	1338 (63%)	206 (46%)
Progression*	1042 (28%)	759 (36%)	240 (54%)
Control group	113 (3%)	12 (1%)	1 (0%)
Kellgren-Lawrence grade in the right knee, n (%)			
0	1049 (28%)	468 (22%)	72 (16%)
1	606 (16%)	305 (14%)	52 (12%)
2	1160 (31%)	681 (32%)	146 (33%)
3	724 (19%)	480 (23%)	129 (29%)
4	235 (6%)	173 (8%)	48 (11%)

*PASE* Physical Activity Scale of the Elderly

*only participants in this subcohort had symptomatic OA in one or both knees at baseline

### Linear change of T_2_ values and cartilage thickness

Baseline values and annual rates of change of MRI-based cartilage biomarkers calculated in individual linear regression models are shown in Table 2. 61 (1.6%) and 62 (1.7%) persons had missing cartilage thickness data (baseline and annual rates of change) in femorotibial and patellar regions, respectively. 91 (2.5%) and 98 (2.7%) persons had missing T_2_ data (baseline and annual rates of change) in femorotibial and patellar regions, respectively. Mean T_2_ values in all regions showed a slight increase from baseline to the 36-month visit, e.g. in MF with 39.0 ± 2.87 ms at baseline and 0.27 ± 0.83 ms annual change. Mean cartilage thickness showed a slight decrease in all regions over the same period, e.g. in MF with 1.86 ± 0.31 mm at baseline and − 0.017 ± 0.047 mm annual change (Fig. 2). Associations of T_2_ values with changes in cartilage thickness over time are shown in Table S4. Longer T_2_ values in LT at baseline were strongly associated with greater annual rates of cartilage thinning. Furthermore, there was a strong relationship between greater annual rates of T_2_ increase and greater annual rates of cartilage thinning in the femoral, lateral tibial, and patellar regions.

**Table 2:** Means and standard deviations of baseline MRI-based cartilage biomarkers and annual rates of change in the right knee (raw values)

	Baseline	Annual change
T2 values [ms]		
Medial Femoral	39.0 ± 2.87	0.27 ± 0.83
Lateral Femoral	35.5 ± 2.74	0.56 ± 0.94
Medial Tibial	30.2 ± 2.19	0.47 ± 0.86
Lateral Tibial	28.4 ± 2.41	0.50 ± 0.98
Patellar	33.3 ± 3.62	0.29 ± 1.73
Cartilage thickness [mm]		
Medial Femoral	1.86 ± 0.31	−0.017 ± 0.047
Lateral Femoral	1.86 ± 0.27	−0.009 ± 0.040
Medial Tibial	1.75 ± 0.27	−0.009 ± 0.030
Lateral Tibial	2.03 ± 0.34	−0.015 ± 0.031
Patellar	2.16 ± 0.41	−0.026 ± 0.046

Baseline values and annual rates of change represent intercepts and slopes, respectively, from individual linear regression models

### Associations between cartilage biomarkers and the presence of knee pain

Associations of T_2_ values and cartilage thickness measured from baseline to 36 months with dichotomized intermittent and constant knee pain at 48 months are shown in Table 3. Knee pain scores were dichotomized to the presence of pain (score > 0). Longer baseline T_2_ values in MF and LF had higher odds for the presence of intermittent knee pain (OR = 1.05 for 1 ms increase; 95% CI = 1.02, 1.08; q = 0.004 for MF and OR = 1.06 for 1 ms increase; 95% CI = 1.03, 1.09; q = 0.002 for LF). Thicker baseline cartilage in PAT had lower odds for the presence of intermittent knee pain (OR = 0.65 for 1 mm increase; 95% CI = 0.53, 0.81; q = 0.002). In other words, thinner baseline cartilage in PAT had higher odds for the presence of intermittent knee pain. Greater annual rates of cartilage thinning in PAT had higher odds for the presence of constant knee pain (OR = 93.4 for 1 mm/y greater rate; 95% CI = 7.66, 1139; q = 0.004).

**Table 3:** Associations between T_2_ values and cartilage thickness and the presence of intermittent and constant pain

	Intermittent Pain	Constant Pain
Baseline T_2_ value [ms]		
Medial Femoral	**1.05 (1.02, 1.08)**	1.02 (0.97, 1.08)
Lateral Femoral	**1.06 (1.03, 1.09)**	0.99 (0.93, 1.05)
Medial Tibial	1.01 (0.98, 1.05)	1.03 (0.96, 1.10)
Lateral Tibial	1.02 (0.99, 1.05)	0.98 (0.92, 1.05)
Patellar	1.01 (0.99, 1.04)	0.94 (0.89, 0.99)
Annual rate of increase in T_2_ value [ms]		
Medial Femoral	1.05 (0.95, 1.17)	1.27 (1.04, 1.55)
Lateral Femoral	1.05 (0.95, 1.16)	1.18 (0.98, 1.42)
Medial Tibial	0.94 (0.84, 1.06)	1.24 (1.01, 1.52)
Lateral Tibial	0.96 (0.87, 1.06)	1.13 (0.98, 1.29)
Patellar	0.99 (0.92, 1.06)	0.87 (0.76, 0.99)
Baseline cartilage thickness [mm]		
Medial Femoral	0.88 (0.68, 1.13)	0.65 (0.41, 1.03)
Lateral Femoral	0.98 (0.73, 1.30)	0.82 (0.47, 1.41)
Medial Tibial	1.13 (0.85, 1.50)	0.49 (0.28, 0.83)
Lateral Tibial	0.76 (0.60, 0.96)	0.64 (0.41, 0.98)
Patellar	**0.65 (0.53, 0.81**)	0.94 (0.63, 1.39)
Annual rate of cartilage thinning [mm]		
Medial Femoral	1.65 (0.37, 7.37)	13.2 (1.02, 171)
Lateral Femoral	0.29 (0.05, 1.76)	6.58 (0.29, 151)
Medial Tibial	0.13 (0.01, 1.31)	13.7 (0.27, 699)
Lateral Tibial	7.67 (0.84, 70.3)	87.8 (3.19, 2415)
Patellar	1.42 (0.32, 6.29)	**93.4 (7.66, 1139)**

Odds ratios (95% CIs) are from logistic regression models adjusting for age, sex, subcohort membership, baseline BMI, and baseline PASE score

Odds reflect the presence of intermittent and constant pain per 1 ms longer baseline T_2_ value, per 1 mm thicker cartilage, per 1 ms greater annual rate of increase in T_2_, and per 1 mm greater annual rate of cartilage thinning

Bold indicates statistical significance at a false discovery rate q < 0.05

The sensitivity analysis yielded similar results adjusting for baseline WOMAC pain instead of subcohort membership, except for the association of cartilage thinning in PAT with constant knee pain that did not reach statistical significance after adjusting for multiple comparisons (Table S1).

### Associations between cartilage biomarkers and knee pain scores

Associations of T_2_ values and cartilage thickness measured from baseline to 36 months with intermittent and constant knee pain scores at 48 months for participants with any intermittent or constant pain, respectively, are shown in Table 4. Thicker baseline cartilage in LT was associated with lower constant knee pain scores (Beta coeff.=−11.8 for 1 mm increase; 95% CI= −19.8, −3.76; q = 0.042). In other words, thinner baseline cartilage in LT was associated with more constant knee pain. Greater annual rates of increase in T_2_ values in MT and LT were associated with higher intermittent knee pain scores (percent change = 8.02 for 1 ms/y greater rate; 95% CI = 2.87, 13.4; q = 0.027 for MT and percent change = 7.85 for 1 ms/y greater rate; 95% CI = 3.39, 12.5; q = 0.009 for LT). Greater annual rates of cartilage thinning in LT were associated with higher intermittent knee pain scores (percent change = 80.2 for 1 mm/y greater rate; 95% CI = 55.9, 91.2; q = 0.003).

**Table 4:** Association between T_2_ values and cartilage thickness and intermittent and constant pain scores in participants with any intermittent or constant pain, respectively

	Intermittent Pain*	Constant Pain
Baseline T_2_ value [ms]		
Medial Femoral	−0.15 (−1.34, 1.06)	−0.17 (−1.29, 0.96)
Lateral Femoral	0.27 (−0.96, 1.51)	−0.51 (−1.62, 0.60)
Medial Tibial	1.31 (−0.26, 2.90)	−0.31 (−1.82, 1.20)
Lateral Tibial	1.05 (−0.29, 2.40)	−0.77 (−2.04, 0.51)
Patellar	0.002 (−1.00, 1.02)	0.16 (−0.72, 1.04)
Annual rate of increase in T_2_ value [ms]		
Medial Femoral	4.68 (0.17, 9.41)	1.92 (−1.96, 5.80)
Lateral Femoral	3.61 (−0.54, 7.94)	0.87 (−2.27, 4.01)
Medial Tibial	**8.02 (2.87**,** 13.4)**	2.66 (−1.35, 6.66)
Lateral Tibial	**7.85 (3.39**,** 12.5)**	1.07 (−2.46, 4.60)
Patellar	1.67 (−1.19, 4.60)	0.91 (−1.38, 3.20)
Baseline cartilage thickness [mm]		
Medial Femoral	−6.70 (−15.9, 3.45)	−5.26 (−13.8, 3.26)
Lateral Femoral	−2.25 (−13.2, 10.0)	−12.1 (−22.4, −1.81)
Medial Tibial	−6.89 (−17.1, 4.62)	−3.19 (−13.3, 6.88)
Lateral Tibial	−10.4 (−18.4, −1.54)	**−11.8 (−19.8**,** −3.76)**
Patellar	−0.20 (−8.50, 8.85)	4.51 (−3.82, 12.8)
Annual rate of cartilage thinning [mm]		
Medial Femoral	54.4 (15.8, 75.3)	0.64 (−3.90, 5.18)
Lateral Femoral	53.4 (4.47, 77.3)	3.25 (−2.00, 8.49)
Medial Tibial	31.7 (−69.5, 72.5)	3.70 (−3.19, 10.6)
Lateral Tibial	80.2 (55.9, 91.2)	1.94 (−5.08, 8.96)
Patellar	25.7 (−43.8, 61.6)	4.64 (0.16, 9.11)

Beta coefficients for intermittent/constant pain (95% CIs) are from linear regression models adjusting for age, sex, subcohort membership, baseline BMI, and baseline PASE score

Beta coefficients reflect the change in constant pain scores per 1 ms longer baseline T_2_ value, per 1 mm thicker cartilage, per 1 ms greater annual rate of increase in T_2_, and per 1 mm greater annual rate of cartilage thinning

*Intermittent pain scores were log-transformed to reduce heteroscedasticity. Thus, change in intermittent pain score is given as $\:percent\:change\:\approx\:100\times\:({e}^{Beta\:coeff.}-1)$ per 1 unit increase in predictor as described above

Models include only participants with any intermittent (*n*=1671) or constant pain (*n*=276), respectively. Bold indicates statistical significance at a false discovery rate q < 0.05

The sensitivity analysis yielded similar results adjusting for baseline WOMAC pain instead of subcohort membership, except for the associations of baseline cartilage thickness in LT with constant pain that did not reach statistical significance after adjusting for multiple comparisons (Table S2).

## Discussion

In this study, we investigated whether MRI-based cartilage biomarkers at baseline and their annual change over 36 months were related to future intermittent and constant knee pain. First, we found that knees with longer femoral T_2_ values at baseline, a surrogate for cartilage degeneration [25], had future intermittent pain. No relationship between T_2_ values and future constant pain was found. Second, knees with thinner patellar cartilage at baseline had future intermittent pain, whereas knees with higher rates of patellar cartilage thinning had future constant pain. Only considering knees with any pain, we found that future intermittent pain increased with greater rates of lateral tibial cartilage thinning and future constant pain increased with thinner lateral tibial cartilage at baseline. All observations were independent of the presence of symptomatic femorotibial OA or risk factors for the development of OA at baseline.

We were able to demonstrate a strong relationship between cartilage loss and knee pain that could be observed in the patellar and lateral tibial regions. A systematic review reported conflicting evidence of the association between cartilage defects and pain. Three of five high-quality studies showed a positive association of cartilage loss with pain [15]. In line with our results, a study of participants with symptomatic knee OA that quantified tibial cartilage volume found a weak association between loss of tibial cartilage and worsening of symptoms [17]. A study of elderly participants with knee OA showed that cartilage morphology assessed using semi-quantitative readings was associated with knee pain [18]. A study of middle-aged women with knee OA using semi-quantitative readings found that full-thickness cartilage defects occurred frequently in painful knee OA [19]. Not paralleled in our results, a study of 718 OAI participants found that the medial femoral condyle showed higher rates of cartilage loss over 1 year in those with frequent knee pain than those with infrequent pain and the least change in those with no pain [20]. More recently, a study of OAI participants with baseline KL-grade 0 knees found a significant association of cartilage full thickness loss with increased knee pain [21]. Another very recent study of a young adult population found that cartilage thickness in medial femorotibial compartments was negatively associated with knee symptoms (sum score of pain, stiffness, and physical dysfunction) [22]. A study measuring knee cartilage thickness change over 12 months found the presence of frequent pain to be a predictor for rapid cartilage thinning and thickening [23].

A systematic review including only asymptomatic uninjured knees concluded that OA features on MRI are common in these knees and are generally associated with age [43]. Further, given pooled prevalence estimates of 19%–43% in older adults without symptoms, structural changes in MRI should be interpreted with caution and targeted therapeutic interventions may not alleviate pain in symptomatic patients. This is in line with our study insofar as we only found patellar cartilage thickness loss linked to the presence of knee pain. Patellofemoral knee pain is considered a clinical entity that is on a continuum with patellofemoral OA and distinct from femorotibial OA [44]. Our statistical models were adjusted for the presence of symptomatic knee OA at baseline (by adjusting for subcohort membership) based on radiographic femorotibial, not patellofemoral OA. This could introduce bias by isolated symptomatic patellofemoral OA at baseline that was not adjusted for. Thus, associations between knee pain and patellar cartilage biomarkers in our study may not be independent of baseline pain.

There is evidence that knee pain is not only a consequence of structural deterioration in OA but also a driver of structural progression. In an inverse study design, positive linear relationships were found between the frequency of knee pain over 1 year and rates of cartilage volume loss over 4 years [45]. Of note, this study found associations with medial and lateral cartilage volume loss, but did not distinguish femoral and tibial regions.

The lateral tibial cartilage seems to play a special role in pain experience as this was the only region where we found correlations between changes in cartilage thickness and pain severity. We observed that the two different pain types related to either baseline cartilage thickness or rate of thinning. Thinner baseline cartilage led to more constant pain. Higher rates of cartilage thinning led to more intermittent pain. The latter could reflect an earlier stage of knee OA with progressive cartilage thickness loss, whereas the former could signify an advanced stage of knee OA with definite cartilage loss. Although no causal inferences can be drawn from our results, they seem to support the concept of two distinct types of pain [12] with intermittent pain representing earlier stages of OA and constant pain manifesting in late-stage OA when organ reserves are depleted [46] and pain sensitization could play a role [47]. We acknowledge that the level of pain in OA patients can be modulated by treatment. However, only a limited number of OAI participants took pain medications as shown in Table S3.

Four patterns of knee pain experience over 4 years were recently described using ICAOP data in the OAI cohort [48]. Membership in less favorable groups that included intermittent and/or constant pain of mild to moderate severity correlated with advanced stage radiographic knee OA (KL-grade 3 and 4) at baseline. Of note, KL-grades 3 and 4 require definite joint space narrowing equivalent to substantial cartilage thickness loss [32]. In this study, associations of baseline radiographic and other predictors with pain patterns in the following 4 years were investigated [48]. In contrast, we studied how linear patterns of change in cartilage imaging biomarkers from baseline to year 3 predicted knee pain at year 4.

Although cartilage cannot directly generate pain, it is assumed that (pre)structural alterations in cartilage result in pain mediated by the synovia and processes at the osteochondral junction where nociceptive stimuli originate [28]. We found that future intermittent knee pain was present in knees with longer femoral T_2_ values at baseline and that pain relatively increased with a steeper increase in tibial T_2_ values over time in those knees. In contrast, future constant knee pain was not influenced by cartilage T_2_ values. T_2_ values of articular cartilage are a surrogate for the integrity of the collagen network where longer values indicate increased free water content and cartilage degeneration [25]. Changes in T_2_ values can be transient due to high strain, as shown in marathon runners [49, 50]. T_2_ changes can precede morphologic changes in the knee cartilage [30, 51] with longer T_2_ values predicting the onset of radiographic tibiofemoral OA [52]. Superficial, not deep, cartilage layer T_2_ values in the medial femorotibial compartment were associated with clinically relevant knee OA progression [53]. Furthermore, T_2_ values correlate with clinical outcome scores following knee cartilage repair [54]. In line with our results, cross-sectional associations between longer femoral T_2_ values and increased KOOS pain were previously reported for the entire OAI cohort [36]. A study of OAI participants with rapid radiographic progression of OA found that all changes in cartilage T_2_ values occurred in the early stages of radiographic OA, while cartilage thickness changes occurred mostly in the later stages [29].

This study has limitations. First, both the exposure (MRI biomarkers) and outcome (future pain) are likely influenced by baseline pain in either direction. ICAOP scores were only available at 48 months, which limited our ability to study the incidence of these types of pain for lack of baseline assessments. Pain patterns including, both, constant and intermittent pain were found to be associated with greater WOMAC pain severity [55]. We decided to adjust for subcohort membership that is based on the presence of symptomatic knee OA at baseline rather than focusing on pain alone. Thereby, we follow the original design of the OAI that aims to study the onset and progression of OA in different subcohorts using common definitions for clinically significant osteoarthritis similar to ACR criteria [31]. Additionally, we conducted a sensitivity analysis adjusting our regression models for baseline WOMAC pain. The sensitivity analysis showed similar results except for associations of cartilage thickness with constant pain that had similar point estimates but only reached the corrected level of statistical significance in models adjusted for subcohort membership. Second, we did not distinguish between deep and superficial cartilage layers that previously showed different changes in T_2_ values associated with OA [52] and disease progression [53]. Third, the requirement for at least two MRI scans for the calculation of regression slopes potentially introduced a selection bias, but only 3.4% of participants had to be excluded. Generalizability of our results is limited to the OAI population of mainly White and Black or African Americans of 45–79 years of age that had no contraindications for MRI. A strength of our study is that we calculated FDR q-values to define statistical significance and reduce type I errors in a multiple testing scenario.

## Conclusions

Changes in femoral cartilage composition were strongly associated with intermittent knee pain typically found in early-stage OA. In contrast, constant knee pain typically found in advanced-stage OA was only related to cartilage thickness loss in tibial and patellar regions. These results support the concept that intermittent pain is characteristic of earlier stages of OA that are paralleled by changes in cartilage composition detectable with MRI.

## Supplementary Information

Supplementary Material 1.

## Data Availability

This article was prepared using a public-use data set of the Osteoarthritis Initiative (OAI) (https://nda.nih.gov/oai/).

## Abbreviations

ACR: American college of rheumatology
BMI: Body mass index
CI: Confidence interval
DESS: Dual-echo in steady state
FDR: False discovery rate
HIPAA: Health insurance portability and accountability act
ICOAP: Intermittent and constant osteoarthritis pain
KL: Kellgren and Lawrence
KOOS: Knee outcomes in osteoarthritis survey
LF: Lateral femoral
LT: Lateral tibial
MF: Medial femoral
MRI: Magnetic resonance imaging
MSME: Multi-slice multi‐echo
MT: Medial tibial
NIAMS: National institute of arthritis and musculoskeletal and skin diseases
OA: Osteoarthritis
OAI: Osteoarthritis initiative
OARSI: Osteoarthritis research society international
OMERACT: Outcome measures in rheumatology
OR: Odds ratio
OSMB: Observational study monitoring board
PASE: Physical activity scale for the elderly
PAT: Patellar
VAS: Visual analog scale
WOMAC: Western ontario and mcmaster universities osteoarthritis index
