# Supplementary material for: Association between constant and intermittent knee pain and T2 values and cartilage thickness: data from the osteoarthritis initiative
Source: Arthritis Res Ther. 2025 Oct 22;27:196. doi: 10.1186/s13075-025-03667-9 (PMC12542445; doi:10.1186/s13075-025-03667-9)
Supplement: Supplementary file 1 — Supplementary Material 1. [file 13075_2025_3667_MOESM1_ESM.docx]

# Supplementary material

Table S 1: Sensitivity analysis; associations between T_2_ values and cartilage thickness and the presence of intermittent and constant pain

|  | Intermittent Pain | Constant Pain |
| --- | --- | --- |
| Baseline T_2_ value [ms] |  |  |
| Medial Femoral | **1.07 (1.04, 1.10)** | 1.05 (0.99, 1.12) |
| Lateral Femoral | **1.07 (1.04, 1.11)** | 1.01 (0.95, 1.07) |
| Medial Tibial | 1.02 (0.99, 1.06) | 1.04 (0.96, 1.12) |
| Lateral Tibial | 1.03 (1.00, 1.07) | 1.00 (0.94, 1.07) |
| Patellar | 1.01 (0.99, 1.04) | 0.95 (0.90, 1.00) |
| Annual rate of increase in T_2_ value [ms] |  |  |
| Medial Femoral | 1.06 (0.95, 1.17) | 1.30 (1.06, 1.60) |
| Lateral Femoral | 1.06 (0.96, 1.17) | 1.17 (0.97, 1.41) |
| Medial Tibial | 0.96 (0.86, 1.07) | 1.26 (1.02, 1.57) |
| Lateral Tibial | 0.95 (0.86, 1.05) | 1.11 (0.97, 1.27) |
| Patellar | 0.99 (0.93, 1.06) | 0.89 (0.78, 1.01) |
| Baseline cartilage thickness [mm] |  |  |
| Medial Femoral | 0.96 (0.74, 1.23) | 0.82 (0.51, 1.33) |
| Lateral Femoral | 1.14 (0.85, 1.52) | 1.17 (0.67, 2.06) |
| Medial Tibial | 1.30 (0.98, 1.74) | 0.66 (0.38, 1.15) |
| Lateral Tibial | 0.77 (0.61, 0.97) | 0.79 (0.50, 1.24) |
| Patellar | **0.62 (0.50, 0.76)** | 0.92 (0.61, 1.38) |
| Annual rate of cartilage thinning [mm] |  |  |
| Medial Femoral | 1.34 (0.30, 6.06) | 5.53 (0.39, 78.3) |
| Lateral Femoral | 0.18 (0.03, 1.10) | 1.12 (0.04, 29.3) |
| Medial Tibial | 0.05 (0.005, 0.53) | 1.05 (0.02, 72.1) |
| Lateral Tibial | 5.86 (0.65, 53.1) | 47.0 (1.41, 1565) |
| Patellar | 0.84 (0.19, 3.78) | 29.6 (2.21, 396) |

Odds ratios (95% CIs) from logistic regression models adjusting for age, sex, baseline WOMAC pain, baseline BMI, and baseline PASE score. Odds reflect the presence of intermittent and constant pain per 1 ms longer baseline T_2_ value, per 1 mm thicker cartilage, per 1 ms greater annual rate of increase in T_2_, and per 1 mm greater annual rate of cartilage thinning. Bold indicates statistical significance at a false discovery rate q<0.05.

Table S 2: Sensitivity analysis; associations between T_2_ values and cartilage thickness and intermittent and constant pain scores in participants with any intermittent or constant pain, respectively

|  | Intermittent Pain* | Constant Pain |
| --- | --- | --- |
| Baseline T_2_ value [ms] |  |  |
| Medial Femoral | 0.44 (-0.71, 1.61) | 0.15 (-0.93, 1.23) |
| Lateral Femoral | 0.91 (-0.27, 2.11) | -0.22 (-1.28, 0.84) |
| Medial Tibial | 1.51 (0.004, 3.04) | -0.10 (-1.53, 1.33) |
| Lateral Tibial | 1.44 (0.16, 2.75) | -0.52 (-1.74, 0.70) |
| Patellar | 0.15 (-0.81, 1.13) | 0.09 (-0.75, 0.92) |
| Annual rate of increase in T_2_ value [ms] |  |  |
| Medial Femoral | 5.28 (0.91, 9.84) | 2.33 (-1.38, 6.04) |
| Lateral Femoral | 4.41 (0.39, 8.59) | 1.18 (-1.83, 4.19) |
| Medial Tibial | **8.56 (3.59, 13.8)** | 3.26 (-0.56, 7.08) |
| Lateral Tibial | **7.30 (3.03, 11.7)** | 1.36 (-2.01, 4.73) |
| Patellar | 1.72 (-1.02, 4.54) | 0.69 (-1.49, 2.87) |
| Baseline cartilage thickness [mm] |  |  |
| Medial Femoral | -3.70 (-12.8, 6.36) | -4.62 (-12.7, 3.48) |
| Lateral Femoral | 1.40 (-9.50, 13.6) | -10.1 (-19.8, -0.33) |
| Medial Tibial | -2.43 (-12.8, 9.16) | -3.42 (-13.0, 6.13) |
| Lateral Tibial | -8.46 (-16.3, 0.15) | -10.4 (-18.1, -2.79) |
| Patellar | -0.67 (-8.57, 7.91) | 1.83 (-5.88, 9.54) |
| Annual rate of cartilage thinning [mm] |  |  |
| Medial Femoral | 51.6 (12.8, 73.1) | -0.58 (-43.8, 42.6) |
| Lateral Femoral | 44.5 (-10.8, 72.2) | 21.6 (-28.8, 71.9) |
| Medial Tibial | 20.8 (-89.9, 66.9) | 23.2 (-42.8, 89.1) |
| Lateral Tibial | **78.4 (53.3, 90.0)** | 6.95 (-60.3, 74.2) |
| Patellar | 2.20 (-84.1, 48.1) | 40.5 (-2.38, 83.4) |

Beta coefficients for intermittent/constant pain (95% CIs) from linear regression models adjusting for age, sex, baseline WOMAC pain, baseline BMI, and baseline PASE score. Beta coefficients reflect the change in constant pain scores per 1 ms longer baseline T_2_ value, per 1 mm thicker cartilage, per 1 ms greater annual rate of increase in T_2_, and per 1 mm greater annual rate of cartilage thinning. *Intermittent pain scores were log-transformed to reduce heteroscedasticity. Thus, change in intermittent pain score is given as $percent change \approx100\times(e^{Beta coeff.}-1)$ per 1 unit increase in predictor as described above. Models include only participants with any intermittent (n=1671) or constant pain (n=276), respectively. Bold indicates statistical significance at a false discovery rate q<0.05.

Table S 3: Baseline use of pain medication stratified by the presence of any intermittent or any constant pain at 48 months

|  | Any intermittent pain | | Any constant pain | |
| --- | --- | --- | --- | --- |
|  | N | % | N | % |
| Used this drug for joint pain or arhtitis more than half the days of the month in the past 30 days: |  |  |  |  |
| Acetaminophen | 136 | 3.6% | 82 | 2.2% |
| Nonprescription NSAIDs | 278 | 7.4% | 153 | 4.0% |
| Prescription NSAIDs | 93 | 2.5% | 48 | 1.3% |
| COXIBs | 82 | 2.2% | 55 | 1.5% |
| Narcotics | 35 | 0.9% | 25 | 0.7% |
| SAMe | 8 | 0.2% | 5 | 0.1% |
| MSM | 86 | 2.3% | 29 | 0.8% |
| Doxycycline | 4 | 0.1% | 1 | 0.0% |

NSAIDs= Non-steroidal anti-inflammatory drugs, COXIBs= Cyclooxygenase-2 inhibitors, SAMe= S-Adenosyl methionine, MSM= Methylsulfonylmethane.

Table S 4: Beta coefficients for annual cartilage thinning rates in the corresponding regions of interest in µm/year

|  | Beta coeff. (95% CI) | P-value |
| --- | --- | --- |
| Baseline T_2_ value [ms] |  |  |
| Medial Femoral | 0.41 (1.01, -0.18) | 0.172 |
| Lateral Femoral | -0.26 (0.30, -0.81) | 0.370 |
| Medial Tibial | 0.05 (0.57, -0.47) | 0.847 |
| Lateral Tibial | **1.34 (1.78, 0.89)** | <0.001 |
| Patellar | 0.37 (0.92, -0.18) | 0.184 |
| Annual rate of increase in T_2_ value [ms] |  |  |
| Medial Femoral | **3.46 (1.18, 5.74)** | 0.003 |
| Lateral Femoral | **6.50 (4.63, 8.38)** | <0.001 |
| Medial Tibial | 1.20 (-0.38, 2.78) | 0.135 |
| Lateral Tibial | **8.89 (7.63, 10.15)** | <0.001 |
| Patellar | **2.04 (0.54, 3.55)** | 0.008 |

Beta coefficients (95% confidence intervals) are from linear regression models adjusting for age, sex, and BMI. Beta coefficients reflect the change in annual rates of cartilage thinning per 1 ms longer baseline T_2_ value and per 1 ms/year greater annual increase in T_2_. Bold indicates statistical significance at p<0.05.
